# *Methylotetracoccus oryzae* Strain C50C1 Is a Novel Type Ib Gammaproteobacterial Methanotroph Adapted to Freshwater Environments

**DOI:** 10.1128/mSphere.00631-18

**Published:** 2019-06-05

**Authors:** Mohammad Ghashghavi, Svetlana E. Belova, Paul L. E. Bodelier, Svetlana N. Dedysh, Martine A. R. Kox, Daan R. Speth, Peter Frenzel, Mike S. M. Jetten, Sebastian Lücker, Claudia Lüke

**Affiliations:** a Department of Microbiology, IWWR, Radboud University, Nijmegen, the Netherlands; b Research Center of Biotechnology of the Russian Academy of Sciences, Winogradski Institute of Microbiology, Moscow, Russia; c Department of Microbial Ecology, Netherlands Institute of Ecology (NIOO-KNAW), Wageningen, the Netherlands; d Max Planck Institute for Terrestrial Microbiology, Marburg, Germany; e Department of Biotechnology, Delft University of Technology, Delft, the Netherlands; f Soehngen Institute of Anaerobic Microbiology, Nijmegen, the Netherlands; National Institute of Advanced Industrial Science and Technology

**Keywords:** methane, aerobic methane oxidation, comparative genomics, paddy field, soil microbiome

## Abstract

Methane-oxidizing microorganisms perform an important role in reducing emissions of the greenhouse gas methane to the atmosphere. To date, known bacterial methanotrophs belong to the *Proteobacteria*, *Verrucomicrobia*, and NC10 phyla. Within the *Proteobacteria* phylum, they can be divided into type Ia, type Ib, and type II methanotrophs. Type Ia and type II are well represented by isolates. Contrastingly, the vast majority of type Ib methanotrophs have not been able to be cultivated so far. Here, we compared the distributions of type Ib lineages in different environments. Whereas the cultivated type Ib methanotrophs (*Methylococcus* and *Methylocaldum*) are found in landfill and upland soils, lineages that are not represented by isolates are mostly dominant in freshwater environments, such as paddy fields and lake sediments. Thus, we observed a clear niche differentiation within type Ib methanotrophs. Our subsequent isolation attempts resulted in obtaining a pure culture of a novel type Ib methanotroph, tentatively named “*Methylotetracoccus oryzae*” C50C1. Strain C50C1 was further characterized to be an obligate methanotroph, containing C_16:1_ω9c as the major membrane phospholipid fatty acid, which has not been found in other methanotrophs. Genome analysis of strain C50C1 showed the presence of two *pmoCAB* operon copies and XoxF5-type methanol dehydrogenase in addition to MxaFI. The genome also contained genes involved in nitrogen and sulfur cycling, but it remains to be demonstrated if and how these help this type Ib methanotroph to adapt to fluctuating environmental conditions in freshwater ecosystems.

**IMPORTANCE** Most of the methane produced on our planet gets naturally oxidized by a group of methanotrophic microorganisms before it reaches the atmosphere. These microorganisms are able to oxidize methane, both aerobically and anaerobically, and use it as their sole energy source. Although methanotrophs have been studied for more than a century, there are still many unknown and uncultivated groups prevalent in various ecosystems. This study focused on the diversity and adaptation of aerobic methane-oxidizing bacteria in different environments by comparing their phenotypic and genotypic properties. We used lab-scale microcosms to create a countergradient of oxygen and methane for preenrichment, followed by classical isolation techniques to obtain methane-oxidizing bacteria from a freshwater environment. This resulted in the discovery and isolation of a novel methanotroph with interesting physiological and genomic properties that could possibly make this bacterium able to cope with fluctuating environmental conditions.

## Abstract (precis)

Most of the methane produced on our planet gets naturally oxidized by a group of methanotrophic microorganisms before it reaches the atmosphere. These microorganisms are able to oxidize methane, both aerobically and anaerobically, and use it as their sole energy source. Although methanotrophs have been studied for more than a century, there are still many unknown and uncultivated groups prevalent in various ecosystems. This study focused on the diversity and adaptation of aerobic methane-oxidizing bacteria in different environments by comparing their phenotypic and genotypic properties. We used lab-scale microcosms to create a countergradient of oxygen and methane for preenrichment, followed by classical isolation techniques to obtain methane-oxidizing bacteria from a freshwater environment. This resulted in the discovery and isolation of a novel methanotroph with interesting physiological and genomic properties that could possibly make this bacterium able to cope with fluctuating environmental conditions.

## INTRODUCTION

Methanotrophs are a functional group of diverse Gram-negative bacteria that are defined by their ability to oxidize methane, which they utilize as a source of carbon and energy (1–3). Since their discovery in 1906 by Soehngen, they are known to play a key role in the global methane cycle through the reduction of methane emissions to the atmosphere (4–6). Aerobic methanotrophs utilize methane via a methane monooxygenase (MMO) that exists in a soluble (sMMO) cytoplasmic- and particulate (pMMO)-membrane-bound form, both of which catalyze the first step of methane oxidation to methanol (2). Methane-oxidizing bacteria (MOB) are ubiquitous in nature and have been found in various environments where oxygen and methane are readily available (1, 7). While most grow best with moderate pHs and temperature ranges, psychrophilic, thermophilic, alkaliphilic, and acidophilic methanotrophs have been isolated as well (reviewed in reference 2).

To date, the best-studied methanotrophs belong to the proteobacterial classes *Alpha*- and *Gammaproteobacteria* (2, 8), but MOB within the phyla *Verrucomicrobia* and NC10 (9–11) were recently discovered, expanding the phylogenetic diversity of MOB. Despite this diversity, MOB have remarkably similar methane oxidation pathways, while incorporating different pathways for carbon fixation. Proteobacterial MOB utilize C_1_ compounds via the ribulose monophosphate (RuMP) or serine pathways (3, 12), while verrucomicrobial MOB and NC10 bacteria use the Calvin cycle (13, 14). After the extensive isolation and characterization of methanotrophs that took place in the 1970s, three types of methanotrophs were defined (15, 16). The strains that incorporated carbon into biomass using the RuMP pathway contained intracytoplasmic membranes as vesicular disks, and monounsaturated hexadecenoic (16:1) signature fatty acids were grouped under type I. Type II strains differed from type I strains by utilizing the serine pathway for carbon fixation, having intracytoplasmic membranes aligned along the periphery of the cell and monounsaturated octadecenoic acid (18:1) as a major membrane lipid (12, 15).

In various studies, an additional group of methanotrophs has been described as type X (17, 18), defined originally based on genomic G+C content and intracytoplasmic membrane organization. This group had characteristics that did not define them under one type, possessing the full RuMP pathway as well as ribulose-1,5-bisphosphate carboxylase, indicative of the Calvin cycle, and at the time were considered to be adapted to higher temperatures. A combination of biochemical and molecular analyses, however, has revealed that type X strains should be reclassified under type I methanotrophs, and this clade is now referred to as type Ib (8). Nonetheless, these classifications do not encompass all isolates, with some having unexpected characteristics. For instance, a type II strain possessing signature membrane lipids that resemble type I methanotrophs (19) and Methylothermus thermalis, a gammaproteobacterium that possesses both 16:0 and 18:1 fatty acids typical for type I and II methanotrophs, respectively (20), have been reported.

Within the last 20 years, the genera containing MOB within the *Proteobacteria* have expanded to 23 (reference 21 and the references therein). With the exception of low-pH peat-adapted *Methylocella* (22) and *Methyloferula* (23), which possess only sMMO, all known methanotrophs encode a pMMO (24). The genes for pMMO (*pmoCAB*) but mainly *pmoA*, encoding pMMO subunit A, have been used to survey the MOB diversity in various ecosystems (25–27). These studies have shown remarkable environmental diversity, even within the comparably well-studied proteobacterial clades. Although within the *Gammaproteobacteria* there have been 12 genera of both type Ia and Ib that contain cultivars, isolates are lacking for the many uncultivated environmental sequence clusters (2).

Type Ib methanotrophs are known to possess a high metabolic diversity (28, 29). However, this diversity is still to be fully explored due to the many clades of environmental sequences lacking any isolate. These sequences cover a vast variety of natural habitats, such as peat, upland and wetland soil, hot springs, lakes, rivers, ground water, and the deep sea, potentially representing highly diverse metabolic capabilities (4, 30–32). The presence of multiple pathways for carbon and nitrogen fixation and assimilation and of both soluble and particulate MMOs makes it difficult to generalize when discussing physiological abilities of type Ib methanotrophs or any other type of MOB (33).

Methylococcus capsulatus is the only well-described type Ib organism, and it has since become the model organism for the entire group (34). However, sequences from this group are found mostly in upland soil (35). Presently, most known type Ib organisms seem to occur in freshwater environments, but only a few isolates have been described. These have a tendency to live very close to a methane source and under oxygen-limited conditions (36, 37). In this study, we isolated a novel type Ib methanotroph, tentatively named “*Methylotetracoccus oryzae*” strain C50C1, from a freshwater ecosystem and performed physiological and genomic characterization. Based on observations from electron microscopy and sequence analyses, it belongs to a novel genus that is widely distributed in paddy fields and lake ecosystems, making it a potential model representative for this group. We, furthermore, compared different physiological aspects of this isolate (habitat distribution, optimum growth temperature and pH, and key enzymatic activities) to those of other known isolates within the type Ib methanotrophs.

## RESULTS AND DISCUSSION

### Isolation of a gammaproteobacterial methanotroph from paddy soil.

Incubation of paddy field soil in a methane/oxygen countergradient microcosm and further purification of enriched bacteria on nitrate mineral salts (NMS) medium resulted in three gammaproteobacterial methanotrophs that were classified as type Ib. One strain (referred to as strain C50C1) was further purified via several transfers in liquid NMS medium until a pure culture was obtained.

**FIG 1:**
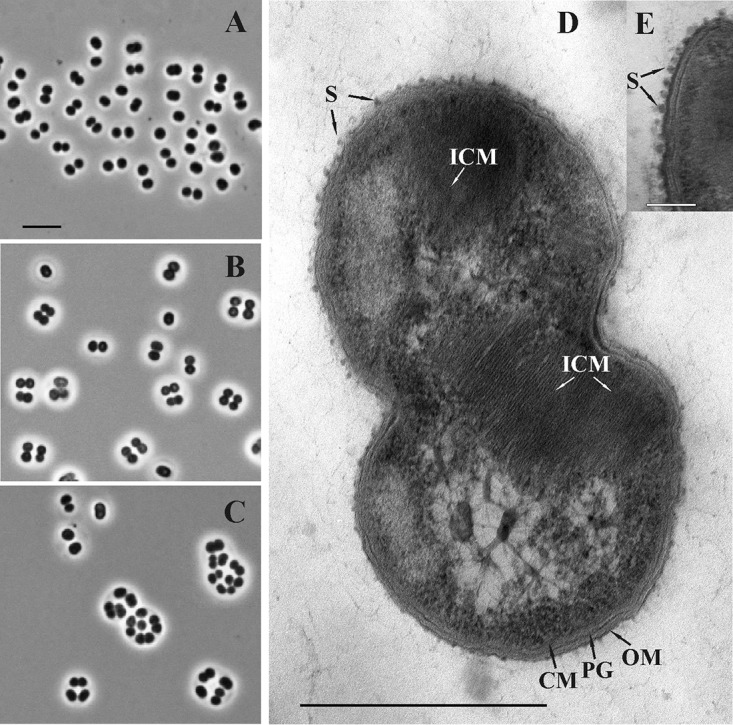
(A, B, C) Phase-contrast micrographs demonstrating the cell morphology of strain C50C1 in 4-, 7-, and 14-day-old cultures. Bar, 5 μm. (D, E) Electron micrograph of an ultrathin section of a cell. ICM, intracytoplasmic membranes; CM, cytoplasmic membrane; OM, outer membrane; PG, peptidoglycan layer; S, S layer. Bars, 0.5 μm (D) and 0.1 μm (E).

Strain C50C1 was represented by Gram-negative and nonmotile cocci or coccoids (1.1 to 1.4 by 1.3 to 1.8 μm in size), which reproduced by binary fission and occurred singly, in pairs, or in tetrads or formed large cell clusters in old (≥2-week) cultures (Fig. 1A to C). Examination of thin-sectioned cells of strain C50C1 revealed a typical Gram-negative structure of the cell wall and the presence of intracytoplasmic membranes, arranged as stacks of vesicular disks (Fig. 1D), which is characteristic of type I methanotrophs. Globular structures apparently representing an S layer were observed on the cell surface (Fig. 1E). Although the presence of S layers is highly characteristic for many type I methanotrophs, including *Methylococcus* species (38), this type of S-layer symmetry has not been reported for any of the previously described methanotrophs.

Strain C50C1 was able to grow only on methane and methanol. Methanol supported growth in the concentration range of 0.1 to 4% (vol/vol); the highest growth rates (doubling time, 21 h) occurred at 3% (vol/vol). No growth was observed on multicarbon compounds. Strain C50C1 grew in the pH range of 4.8 to 8.3, with the optimum at pH 6.8 to 7.5. The temperature range for growth was 4 to 30°C, with the optimum at 18 to 25°C. The doubling time on methane and methanol under optimal growth conditions was 16 and 21 h, respectively. Strain C50C1 was highly sensitive to salt stress and growth was inhibited at NaCl concentrations above 0.3% (wt/vol).

**FIG 2:**
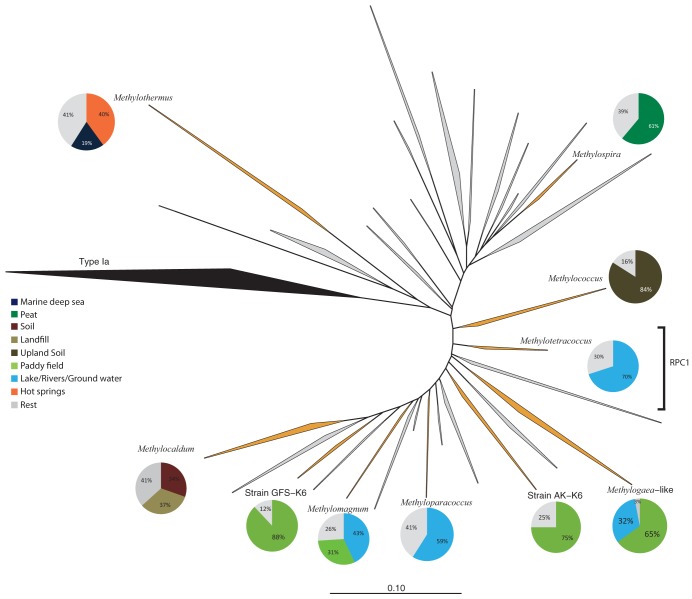
Phylogenetic inference of methane monooxygenase (PmoA) protein sequences of type Ib methanotrophs. The tree is constructed using ARB’s neighbor-joining method. Type Ia sequences were used as the outgroup. Clades in orange are represented by isolates and clades in gray by environmental sequences only. All clusters that contain isolates are accompanied by a pie chart, with colors representing the environments to which the majority of sequences belong. RPC1, rice paddy cluster 1. The bar indicates 0.1 substitution per amino acid position.

Based on 16S rRNA and *pmoA* gene-based phylogeny, strain C50C1 could be classified as type Ib methanotroph affiliated with rice paddy cluster 1 (RPC1) (Fig. 2). RPC1 forms a monophyletic lineage, containing *pmoA* sequences that were mostly retrieved from freshwater environments such as lakes, groundwater and paddy fields (25, 39, 40). So far, few members of type Ib methanotrophs have been characterized, resulting in the description of five genera. However, most clusters contain environmental sequences only and lack cultured representatives (Fig. 2). Closest cultivated relatives of strain C50C1 include Methylococcus capsulatus, Methylocaldum gracile and Methyloparacoccus murrellii (94% 16S rRNA gene identity to each species and 92% amino acid identity to the PmoA of *M. capsulatus*).

### Phenotype and growth characteristics of strain C50C1.

**FIG 3:**
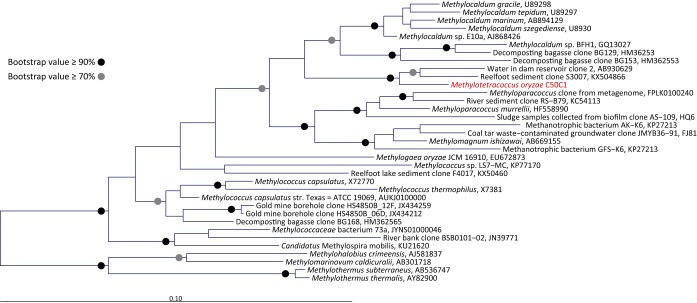
16S rRNA gene-based phylogenetic analysis of a subgroup of closely related type Ib methanotrophs to strain C50C1 (in red), including isolates and environmental clones. Selected members of the *Methylothermaceae* were used to root the tree. The bar indicates 0.1 substitution per nucleotide position.

We made a phenotypic comparison between strain C50C1 and other type Ib isolates (Table 1). C50C1 grows on methane and methanol as sole energy sources (Table S2), is able to fix N_2_ (see Fig. S1 in the supplemental material), and grows at temperatures between 4 and 30°C, which is a much larger range than those of other characterized type Ib methanotrophs (Table 1). Similar to other MOB, it prefers pH values between 6 and 8 and is sensitive to 0.3% NaCl. Major phospholipid-derived fatty acids (PLFAs) in strain C50C1 are C_16:1_ω9c, C_16:1_ω7c, and C_16:0_. C_16:1_ω9c is highly unusual for type Ib methanotrophs, but small amounts have also been detected in *Methylogaea* and *Methyloparacoccus* (Table 1; Table S3). Large amounts of this PLFAs have so far been detected only in MOB belonging to *Alphaproteobacteria* (39), and its presence in strain C50C1 gives it a specific signature. The recently described Methyloterricola oryzae belonging to RPC1 possesses mainly C_16:0_, C_16:1_ω6c, and C_16:1_ω7c, typical of type Ib methanotrophs (41). Based on the complete PLFA profile, however, C50C1 is most closely related to Methyloterricola oryzae, strengthening its placement in RCP1 (Fig. S2). Furthermore, both PmoA (Fig. 2) and 16S rRNA gene-based phylogeny (Fig. 3) show a clear affiliation of strain C50C1 with the type Ib MOB.

**TABLE 1:** Comparison of strain C50C1 growth characteristics to those of other type Ib methanotrophs

Character- istic(s)	Result for^*a*^:								
	*Methylo-* *tetracoccus* *oryzae* C50C1	*Methylo-* *paracoccus* (2 strains)	*Methylo-* *coccus* *capsulatus* (2 strains)	*Methylo-* *caldum* (4 strains)	Strain GFS-K6	*Methyl-* *ogaea* *oryzae*	*Methylo-* *magnum*	Strain AK-K6	*Methylo-* *spira* cluster
Isolation source, country(ies)	Rice field, Italy	Pond water, South Africa and Japan	Thermal bath water, UK	Marine sediment	Terrestrial methane seep pond sediments, Bangladesh	Rice field, Uruguay	Rice fields, Bangladesh and Japan	Warm spring sediments, Armenia	Acidic sphagnum peat bog, Russia
PmoA cluster	Freshwater sediment 2 (RPC1)	Freshwater sediment 2 (RPCs)	*Methylo-* *coccus*-like	*Methylo-* *caldum*-like	*Methylo-* *coccaceae* family	JRP-4	*Methylo-* *coccus*- *Methylo-* *caldum*- *Methylo-* *paracoccus*- *Methylo-* *gaea* clade	*Methylo-* *coccaceae* family	OSC
Major habitat	Freshwater lake	Freshwater lake	Meadow/ shrubs	Soil	Rhizosphere/ root	Paddy field	Lake sediment/ soil	Paddy field	Peat
Growth temp (°C) range, optimum	4–30	20–37, 25–33	28–55, 37–50	20–62	8–35, 25–28	20–37, 30–35	20–37, 31–33	8–35, 25–28	8–25, 14–25
pH range, optimum	6–8	5.8–9, 6.3–6.8	5.5–9.0, ND	5–9, 6–8	5.0–7.5, 6.4–7.0	5–8, 6.5–6.8	5.5–9.0, 6.8–7.4	5.0–7.5, 6.4–7.0	4.2–6.0, 6.0–6.5
Tolerence to 1% NaCl	No	No	Yes	ND	No	No	No	No	No
Key enzyme activities of sMMO, nitrogenase, RubisCO	–, +, –	–, –, –	+, +, +	–, –, +	–, +, +	–, –, –	+, –, –	–, +, +	–, +, +
Cell morph- ology	Cocci	Cocci	Cocci, rods	Rods, pleomorphic	Rods	Curved rods	Rods	Rods	Curved rods (spiral)
Motility	None	None	Variable	Yes	None	None	Yes	None	Yes
Major fatty acid(s)	C16:1ω9c, C16:1ω7c, C16:0	C16:1ω7c*	C16:0, C16:1ω7c*	ND	C16:1ω7c	C16:0	C14:0, C16:0, C16:1ω7c*	C16:1ω7c	ND
Cell size (μm)	1.1–1.4 by 1.3–1.8	0.8–1.5	0.8–1.5 by 1.0–1.5	0.6–1.2 by 1.0–1.8	1.5–2.2 0.5–1.5	0.5–0.7 by 2.0–2.2	1.5–2.0 by 2.0–4.0	1.5–2.2 by 0.5–1.5	1.0–1.5 by 2.0–2.5
Pigmenta- tion	White to brown	White	White to brown	Brown	White	White	White	White	ND
Forma- tion of:									
Cysts	–	–	+	+	–	–	+	–	–
Chains	+	–	+	+	–	–	–	–	–
DNA G+C content (mol%)	62.77	65.6	59–66	56.5–57.2	ND	63.1	64.1	ND	ND
Reference(s)	Current study	Hoefman et al. (80)	Bowman et al. (8)	Takeuchi et al., (43), Bodrossy et al. (42)	Islam et al. (37)	Geymonat et al. (81)	Islam et al. (37), Khalifa et al. (47)	Islam et al. (37)	Danilova et al. (36)

a OSC, organic soil cluster; ND, not determined.

10.1128/mSphere.00631-18.1 FIG S1 Growth dynamics of strain C50C1 in 5-fold-diluted ammonium-containing (circles) or nitrogen-free (squares) mineral salts medium (AMS and MS, respectively) under atmospheric (open symbols) and low (solid symbols) O_2_ levels. Optical density was measured at 600 nm. Arrows represent O_2_ replenishment. Download FIG S1, PDF file, 0.04 MB. Copyright © 2019 Ghashghavi et al. 2019 Ghashghavi et al. This content is distributed under the terms of the Creative Commons Attribution 4.0 International license.

10.1128/mSphere.00631-18.2 FIG S2 PLFA profiles of type I and verrucomicrobial MOB compared by nonmetric multiscale dimensional scaling (NMDS) of PLFA profiles of methanotroph cultures (expressed as percentages of total PLFA content). The two-dimensional distances between samples in the NMDS plot show the relative similarity between samples. The closest matching PLFA profiles are indicated with a minimum spanning tree analysis displaying the shortest distance (i.e., similarity) to connect all PLFA profiles, which results in relating a profile to its nearest neighbor for every sample. The analyses were carried out with the software PAST. Black dots, type Ia; blue squares, type Ib; red triangles, *Methylothermaceae* (type Ic); green dots, *Methylacidiphilaceae* (type III). Download FIG S2, PDF file, 0.3 MB. Copyright © 2019 Ghashghavi et al. 2019 Ghashghavi et al. This content is distributed under the terms of the Creative Commons Attribution 4.0 International license.

### Diversity and ecological niches of type Ib methanotrophs.

To gain an overview of the diversity and habitat preferences of cultivated and uncultivated type Ib methanotrophs, we performed a phylogenetic analysis of approximately 2,800 publicly available *pmoA* sequences from various environments. We classified the habitat information into eight environmental categories and compared the *pmoA* diversity to the environmental origins of the sequences (Fig. 2). Sequences could be grouped into 32 major sequence clusters. For a long time, only the genera *Methylococcus* and *Methylocaldum* were represented by isolates; however, recently several additional type Ib methanotrophs were obtained in pure culture (Fig. 2; Table 1 and the references within). *Methylomagnum*, *Methylogaea*, and strains SK-K6 and GFS-K6 all belong to clusters containing environmental sequences derived mainly from paddy fields. These isolates grow in similar pH ranges, but *Methylogaea* and *Methylomagnum* possess a slightly higher optimum growth temperature of 30 to 35°C.

*Methyloparacoccus* and the tentatively named *Methylotetracoccus* clades have most sequences derived from freshwater ecosystems. Since these strains have been isolated from similar environments, their growth parameters and genome-inferred physiological capabilities are highly similar. Contrastingly, both *Methylococcus* and *Methylocaldum* have been isolated from sources that differ from the major habitat of their respective sequence clade, based on environmental sequences. The former was isolated from a Roman thermal bath, the latter from marine sediment (7, 42, 43). Lastly, “*Candidatus* Methylospira mobilis” appears to be an accurate representative for its clade of mainly peat-derived environmental clones, as it is adapted to acidic conditions (36). Although type Ib MOB have shown to be diverse with regard to their environmental adaptability, they seem to play a very minor role in marine ecosystems, where most sequences belong to type Ia.

### Genome sequencing of strain C50C1.

To gain further insights into the metabolic potential of strain C50C1, we sequenced and analyzed its genome. Assembly and binning resulted in a 4.83-Mbp draft genome consisting of 42 contigs longer than 1 kb. Based on single-copy marker gene analysis, the genome was predicted to be 99.1% complete, with 3.3% contamination. The overall G+C content is 63%. In total, the genome was predicted to contain 4,302 protein coding sequences (CDSs) and one copy of the rRNA operon. Genome size and G+C content are comparable to those of the four other sequenced type Ib methanotrophs, which range from 3.3 to 5 Mbp and 57% to 63%, respectively (Table 1). The rRNA operon copy numbers in bacterial genomes can vary from 1 to as many as 15 copies, and a correlation of copy number with resource availability has been hypothesized (44). Most other type Ib genomes also harbor only one copy, with the exception of Methylococcus capsulatus Bath, which contains two (34). Thus, MOB appear not to be in need of multiple rRNA copies for rapid adaptation to substrate availability, but this requires further analyses once more genomes of type Ib and other types of methanotrophs are sequenced.

### Methane oxidation.

**FIG 4:**
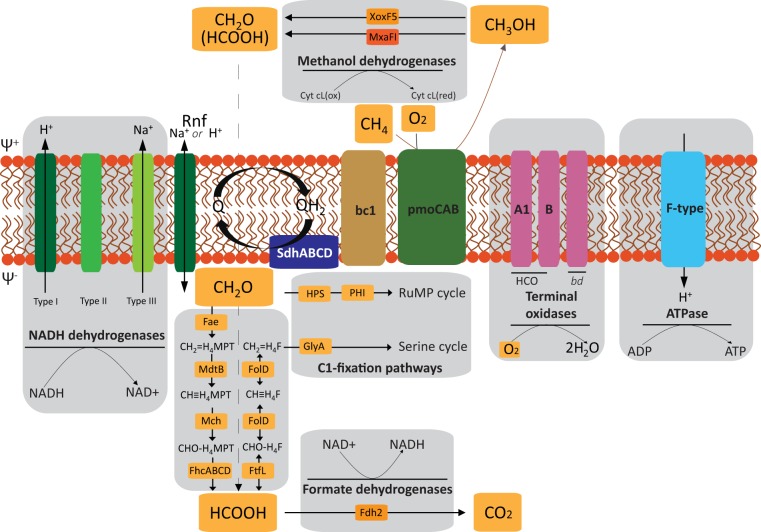
Predicted energy metabolism of strain C50C1. SdhABCD, succinate dehydrogenase; Rnf, NAD-ferredoxin reductase; Q, quinone; bc1, cytochrome *bc*_1_ complex; Fae, formaldehyde-activating enzyme; Mch, methenyl-H_4_MPT cyclohydrolase; FhcABCD, formyltransferase/hydrolase complex; FtfL, formate-tetrahydrofolate ligase; FolD, methylene-H_4_F dehydrogenase/cyclohydrolase; HPS, 3-hexulose-6-phosphate synthase; PHI, 6-phospho-3-hexuloisomerase; GlyA, serine hydroxymethyltransferase.

Based on the genomic information, the metabolic pathways for methane oxidation and energy conservation in strain C50C1 were reconstructed (Fig. 4). The genome includes two copies of the *pmoCAB* operon, encoding the membrane-bound pMMO, and four additional copies of *pmoC*, which are scattered throughout the genome. However, none of the two *pmoCAB* operons encodes the high-affinity pMMO-2 isoenzyme described in *Alphaproteobacteria*, which has been shown to be responsible for oxidation of methane at low mixing ratios (45). Since the concentrations of CH_4_ and O_2_ to which strain C50C1 would be exposed in its natural environment are not comparable to the ones experienced by atmospheric methane oxidizers, possessing a high-affinity pMMO would not necessarily be an advantage in a wetland. Neither the distinct *pmoABC* operon encoding the so-called pXMO (46) nor genes for the sMMO were identified in the genome, although the latter have been found in Methylococcus capsulatus (8) and in several *Methylomagnum* strains (37, 47) (Table 1 and references therein). According to recent studies, sMMO seems not to play a role in methanotrophy in paddy fields, as it was found to be absent in all rice field isolates, and PCR-based studies detected only *mmoX* genes related to *Methylocystis*/*Methylosinus* species (48).

### Methanol and formaldehyde oxidation.

For the subsequent oxidation of methanol to formaldehyde, the C50C1 genome encoded both the lanthanide-dependent XoxF5-type (49, 50) and the calcium-dependent MxaFI-type methanol dehydrogenase (MDH). The XoxF5-type MDH has been shown to have a higher affinity than MxaFI and, unlike the MxaFI-type enzyme, to directly convert methanol to formate in *Methylacidiphilum fumariolicum* SolV, which lacks a dedicated formaldehyde dehydrogenase (51, 52). However, XoxF-type enzymes were also shown to efficiently oxidize formaldehyde (53). In accordance with the dependency of XoxF-type MDHs on pyrroloquinoline quinone (PQQ), strain C50C1 also bears genes for PQQ biosynthesis. Electrons from the oxidation of methanol are transferred to cytochrome *c*_L_, which serves as the primary electron acceptor for MDH. In the periplasm, cytochrome *c*_L_ is oxidized, and the electrons end up at typical membrane-bound terminal oxidases by way of class I *c*-type cytochromes (1).

Most of the reducing equivalents required for the metabolism of methane are produced by the oxidation of formaldehyde (3, 54). Formaldehyde is an important intermediate, as it forms the branching point for anabolic carbon fixation via the serine or RuMP cycle and catabolic substrate oxidation to CO_2_. However, this compound also is highly toxic, and its production and consumption consequently need to be tightly regulated (55).

A variety of enzymes have been shown to catalyze formaldehyde oxidation. Based on their electron acceptor, they can be grouped into nicotinamide adenine dinucleotide phosphate [NAD(P)^+^]-dependent and dye (cytochrome)-linked formaldehyde dehydrogenases (FalDH). Based on the genomic data, strain C50C1 possesses a homolog (74% amino acid identity) to a membrane-associated dye-linked PQQ-dependent FalDH putatively catalyzing formaldehyde oxidation. This enzyme in Methylococcus capsulatus Bath has been characterized (56) and was shown to be a member of the sulfide:quinone oxidoreductase enzyme family. Under high-copper growth conditions, this enzyme was found to be the major formaldehyde dehydrogenase. Additional homologs are present in *Methylocaldum* and *Methylohalobius* with, however, much lower identity (≤40%) and potentially different functions within the sulfide:quinone oxidoreductase family. C50C1 is lacking homologs of *S*-(hydroxymethyl) glutathione dehydrogenase (EC 1.1.1.284), which provides an alternative route from formaldehyde to formate in all other type Ib MOB.

Like other type Ib species, C50C1 has tetrahydrofolate (H_4_F) and 5,6,7,8-tetrahydromethanopterin (H_4_MPT)-linked C_1_ carrier pathways. H_4_MPT is the archaeal analogue of H_4_F and can transfer formyl, methenyl, methylene, and methyl groups (57). These two pathways were regarded as redundant. However, more recent observations have shown that formate might be a branching point for anabolic and catabolic reactions making these two pathways function in parallel (58). The generation of methylene H_4_F and its subsequent entry to the serine pathway is done through direct condensation of formaldehyde with H_4_F. Alternatively, methylene H_4_F can be formed from formate in the tetrahydromethanopterin pathway from H_4_MPT. The latter seems to occur in a facultative methylotrophic, non-methane-oxidizing *Methylobacterium* (59), thus making it likely to occur in strain C50C1 as well. In contrast to *Methylobacterium*, C50C1, furthermore, possesses FolD, a bifunctional methylene-H_4_F dehydrogenase and methenyl-H_4_F cyclohydrolase instead of the usual *mtdA* and *fch* gene pair, encoding enzymes catalyzing the separate reactions, respectively. In Methylobacterium chloromethanicum CM4, FolD has been shown to be specifically involved in dissimilation of the methyl-H_4_F (60). Although this process varies within MOB, all type Ib genomes analyzed to date with the exception of strain C50C1 encode the MtdA/Fch couple and lack FolD.

### Formate oxidation.

In Methylococcus capsulatus Bath and Methylobacterium extorquens, two isoenzymes have been characterized to be involved in formate oxidation (61, 62). The first of these formate dehydrogenases (FDH-1) has been characterized as a tungsten-containing enzyme in M. extorquens and is arranged in a *fdhABC* gene cluster (61). While this enzyme has been identified in Methylococcus capsulatus Bath and *M. capsulatus* Texas, it is not present in other type Ib species, including strain C50C1. Contrastingly, the second FDH-2 is a molybdenum (Mo)-depending enzyme encoded by the *fdhCBAD* gene cluster. This enzyme is found in all other type Ib organisms, including strain C50C1, making it much more widespread than its tungsten-containing counterpart. In general, tungsten enzymes seem to be present mostly in anaerobic microbes, which may be a direct result of its availability and its higher redox properties than those of Mo in anoxic ecosystems (63). Functionally speaking, the two FDHs are virtually identical when their respective cofactor is present (61).

### Energy conservation and respiration.

The draft genome of strain C50C1 encodes a complete electron transport chain, including a proton or sodium ion-translocating NAD-ferredoxin reductase (Rnf) complex, NADH:ubiquinone reductases (H^+^- and Na^+^-transporting types; complex I), succinate dehydrogenase (complex II), cytochrome *bc*_1_ complex enzymes (complex III), quinone-reducing cytochrome *bd*-type enzymes, and putatively cytochrome *c*-reducing heme-copper terminal oxidases (HCO; complex IV) and a F_o_F_1_-type ATPase (complex V) (Fig. 4).

The Rnf (*Rhodobacter* nitrogen fixation) complex is a novel ion-motive electron transport chain found in phylogenetically diverse prokaryotes. In Acetobacterium woodii, the Rnf complex catalyzes oxidation of Fd_red_, with concomitant reduction of NAD^+^ (64). The soluble B subunit (RnfB) of the complex is proposed to be the entry point for electrons from reduced ferredoxin. The C subunit (RnfC) mediates NADH reduction, thus serving as exit point of electrons. The free energy of this reaction is conserved in the electrogenic transport of protons or sodium ions across the membrane, thus establishing an electrochemical potential (64). The genomes of *Methylobacter* and *Methylotenera* encode this complex as well (65). Complex I transfers electrons from NADH into the quinone pool, which is coupled with the translocation of four protons across the inner membrane, further contributing to the formation of a proton motive force (*pmf*) that can be used to synthesize ATP by complex V. Complex II links the tricarboxylic acid (TCA) cycle to the respiratory chain by transferring the electrons derived from succinate oxidation into the quinone pool.

Previous studies have indicated that pMMO also is coupled to the electron transport chain at the level of quinone, with inhibitor studies providing additional evidence of this link (reference 56 and references therein). The oxidation of methane by the pMMO requires the additional activation by oxygen. As one oxygen atom of O_2_ is reduced to H_2_O and the second is incorporated into methane to form methanol, this results in a net consumption of two electrons per methane oxidized. Electrons from the subsequent oxidation of methanol or formaldehyde either end up in a membrane-bound class I *c*-type oxidase or directly enter into the quinone pool, respectively. The reduced quinol then transfers the electrons to the cytochrome *bc*_1_ complex, where the reduction of cytochrome *c* is linked to formation of *pmf* via the so-called Q-cycle. Complex IV finally uses the electrons obtained from cytochrome *c* to reduce O_2_ to H_2_O. This reaction is also linked to active translocation of protons, thus contributing to *pmf*.

The genome of strain C50C1 contains all of the subunits of two members of the HCO superfamily, encoding one A-family and one B-family terminal oxidase. B-family enzymes have been shown to be adapted to lower concentrations of oxygen than those of the A-family, resulting in a higher affinity for O_2_ but fewer protons pumped per electron (66). Possession of both A- and B-family HCO types may allow strain C50C1 to respire using a wide range of oxygen concentrations. This is further supported by the presence of a cytochrome *bd* oxidase, a respiratory quinol:O_2_ oxidoreductase with a very high O_2_ affinity (67). However, enzymes of the *bd* oxidase family conserve less energy than HCOs, as they derive electrons for O_2_ reduction directly from quinol and lack conserved channels for proton pumping, thus bypassing energy conservation at complexes III and IV (66, 67).

### C_1_ fixation and nitrogen and sulfur metabolism.

Fixation of carbon and subsequent assimilation of formaldehyde occurs through the RuMP pathway in strain C50C1, which is typical for type Ib methanotrophs. Additionally, strain C50C1 encodes the serine cycle enzymes serine hydroxymethyl transferase (GlyA), phosphoenolpyruvate (PEP) carboxylase (Ppc), and malate dehydrogenase (Mdh). PEP carboxylase, which is a key enzyme of the serine cycle, is missing in both the *Methylococcus* and *Methylocaldum* genera. The PEP carboxylase encoded by C50C1 belongs to the nonregulated group of PEP carboxylases (68) whose activity is not controlled by intermediates of the TCA cycle or glycolysis/gluconeogenesis (69). Whether these additional enzymes give strain C50C1 an advantage over other type Ib enzymes remains to be investigated. Furthermore, all the enzymes for gluconeogenesis, the TCA cycle, and the nonoxidative pentose phosphate pathway are encoded in strain C50C1’s genome. Unlike with Methylocaldum marinum (43), Methylococcus capsulatus Bath (70), and strain GFS-K6 (37), ribulose-1,5-bisphosphate carboxylase/oxygenase is not encoded in the genome of strain C50C1 (Table 1).

A possible side reaction of the pMMO in MOB is the oxidation of ammonia to hydroxylamine (NH_2_OH). Subsequently, hydroxylamine is detoxified to produce nitrite and nitrous oxide (N_2_O), apparently without linking this reaction to energy conservation (71). Strain C50C1 possesses genes encoding cytochrome *cd*_1_ nitrite reductase (NIR), an NnrS protein involved in response to nitric oxide (NO), NO reductase (NOR), and lastly a NnrU family protein required for NIR and NOR expression. However, hydroxylamine oxidoreductase (HAO) or hydroxylamine reductase is missing from the genome of strain C50C1. As in other MOB, no chemolithotrophic growth was observed on ammonium in strain C50C1, and the apparent lack of hydroxylamine-detoxifying enzymes might contribute to an inability to cope with nitrogen stress caused by nitrification intermediates. However, it has been reported that *M. denitrificans* strain FJG1 under extreme hypoxia couples CH_4_ oxidation to nitrate reduction (72), which may be an explanation for the presence of denitrification genes in strain C50C1.

For nitrogen uptake and assimilation, strain C50C1 encodes three AmtB-type ammonium transporters, a NarK-type nitrate transporter, and assimilatory nitrate and nitrite reductases (encoded by *napA* and *nirBD*). Furthermore, the genome contained all genes for an active nitrogenase for growth under nitrogen-fixing conditions. These include two copies of the dinitrogenase subunits NifD and NifK, the dinitrogenase reductase NifH, as well as the Nif-specific regulatory protein NifA, two copies of the FeMo cofactor biosynthesis protein NifB, the cysteine desulfurase NifS, and the nitrogenase-stabilizing/protective protein NifW.

Like other methanotrophs, such as Methylosarcina lacus and Methylocaldum szegediense, strain C50C1 possesses the full *soxYZ* operon for sulfur oxidation along with the sulfite dehydrogenase SoxD and the sulfur oxidation molybdopterin protein SoxC. However, whether this genomic potential corresponds to an environmental relevance of strain C50C1 in the sulfur cycle remains to be investigated.

### Description of *Methylotetracoccus* gen. nov.

*Methylotetracoccus* [Me.thy.lo.tet.ra.coc′cus]. N.L. n. methylum (from French me′thyle), the methyl group; N.L. pref. methylo, pertaining to the methyl radical; N.L. masc. subst. from Gr. adj. *tetra*, four; N.L. masc. n. coccus (from Gr. n. *kokkos*), a grain or berry; N.L. masc. n. *Methylotetracoccus*, referring to a methyl-using organism with tetrad-forming coccoid cells.

Gram-stain negative, nonmotile cocci or coccoids, which reproduce by binary fission and occur singly, in pairs, or in tetrads or form large cell clusters in old cultures. Cells contain intracytoplasmic membranes, arranged as stacks of vesicular disks. Strictly aerobic, neutrophilic, mesophilic, and nonthermotolerant. Members of the genus are obligate utilizers of C_1_ compounds, such as methane and methanol. Methane is oxidized by pMMO, with sMMO and pXMO being absent. Cells are capable of dinitrogen fixation. The major PLFAs are C_16:1_ω9c, C_16:1_ω7c, and C_16:0_. The most closely related genera are *Methyloparacoccus*, *Methylocaldum*, and *Methylomagnum* within the family *Methylococcaceae* in the class *Gammaproteobacteria*. Known habitats are freshwater ecosystems, such as paddy fields and lake sediments.

### Description of *Methylotetracoccus oryzae* sp. nov.

*Methylotetracoccus oryzae* (O′ryzae N.L. masc. adj. *oryzae*, pertaining to a paddy field).

Description is as for the genus, with the following amendments. Cells are 1.1 to 1.4 μm wide and 1.3 to 1.8 μm long. Growth occurs only on methane and methanol. Methanol supports growth in the range of concentrations of 0.1 to 4% (vol/vol); the highest growth rates with specific generation times of 0.033 h^−1^ (doubling time, 21 h) are observed at 3% (vol/vol). Optimal growth occurs at 18 to 25°C and pH 6.8 to 7.5. Highly sensitive to salt stress; growth is inhibited at NaCl concentrations above 0.3% (wt/vol). The type strain C50C1 was isolated from a paddy field in Cixi, Zhejiang Province, China. The G+C content of the type strain is 63 mol% (genome sequence).

### Conclusions.

In this study, we isolated a novel type Ib methanotroph that can serve as a representative organism for the type Ib freshwater lineage. We report the high-quality draft genome of strain C50C1, which can help design further research to study the role of these MOB in the environment. Based on growth experiments along with genomic data, C50C1 seems to be an obligate methanotroph able to fix nitrogen. The draft genome indicates a potential for metabolic flexibility, with genetic modularity, including multiple methanol dehydrogenases, several pathways for formaldehyde oxidation, all enzymes of one and several enzymes of another pathway for C_1_ fixation, and several terminal oxidases. These genomic potentials may allow strain C50C1 to adapt to various environmental conditions, as already seen in its growth temperature range. The potential for sulfur oxidation within strain C50C1 and its environmental relevance need to be further investigated.

## MATERIALS AND METHODS

### Enrichment conditions and isolation approach.

Enrichments of methane-oxidizing bacteria were started from a paddy field soil sample in Cixi, Zhejiang Province, China (N30°11.066′; E121°21.351′). Soil characteristics and the sampling procedure are described in detail elsewhere (73). Preenrichment was carried out for 14 days in gradient microcosms supplied with 15% methane from the bottom compartment and ambient air from the top (74). After preincubation, the soil was harvested, diluted in nitrate mineral salts (NMS) medium (17) (see Table S1 in the supplemental material), and plated onto solid NMS medium containing 2% agarose. Plates were incubated in air-tight jars supplemented with ambient air and 20% methane. Selected colonies were streaked onto fresh plates to obtain single colonies. The latter, however, were composed not only of methanotrophic bacteria but also of satellite heterotrophic microorganisms. Selected colonies that contained the lowest number of satellite cells were picked and used to inoculate 30-ml serum vials containing 10 ml of 2-fold-diluted NMS medium together with 20 μl of trace element solution I and solution II (Table S1). After inoculation, the vials were sealed with rubber septa, and methane was added aseptically to attain a final mixing ratio of approximately 20% (vol/vol). The inoculated vials were then incubated at 24°C and 100 rpm. The cultures were examined by phase-contrast microscopy, and if morphologically uniform, the cells were transferred to fresh medium and grown again under the same growth conditions. This process of serial dilutions was repeated over 6 months until the target isolate, designated strain C50C1, was obtained in a pure culture. Once isolated, this methanotroph was maintained in 2-fold-diluted NMS medium and was subcultured at 4-week intervals.

10.1128/mSphere.00631-18.3 TABLE S1 Compositions of AMS, NMS, and MS media (as described by Whittenbury et al. [16]) and trace element solutions. Download Table S1, DOCX file, 0.02 MB. Copyright © 2019 Ghashghavi et al. 2019 Ghashghavi et al. This content is distributed under the terms of the Creative Commons Attribution 4.0 International license.

10.1128/mSphere.00631-18.4 TABLE S2 Substrate utilization pattern of strain C50C1. Download Table S2, DOCX file, 0.02 MB. Copyright © 2019 Ghashghavi et al. 2019 Ghashghavi et al. This content is distributed under the terms of the Creative Commons Attribution 4.0 International license.

10.1128/mSphere.00631-18.5 TABLE S3 Phospholipid fatty acid (PLFA) profiles of strain C50C1. Download Table S3, DOCX file, 0.02 MB. Copyright © 2019 Ghashghavi et al. 2019 Ghashghavi et al. This content is distributed under the terms of the Creative Commons Attribution 4.0 International license.

### Phase-contrast and electron microscopy.

Morphological observations and cell size measurements were made with a Zeiss Axioplan 2 microscope and AxioVision 4.2 software (Zeiss, Jena, Germany). Cell morphology was examined by using batch cultures grown to the early-exponential, late-exponential, and stationary growth phases. For preparation of ultrathin sections, cells of the exponentially growing culture of strain C50C1 were collected by centrifugation and prefixed with 1.5% (wt/vol) glutaraldehyde in 0.05 M cacodylate buffer (pH 6.5) for 1 h at 4°C and then fixed with 1% (wt/vol) OsO_4_ in the same buffer for 4 h at 20°C. After dehydration in an ethanol series, the samples were embedded into Epon 812 epoxy resin. Thin sections were cut on an LKB-4800 microtome (LKB-Produkter AB, Stockholm, Sweden) and stained with 3% (wt/vol) uranyl acetate in 70% (vol/vol) ethanol. The specimen samples were examined with a JEM-100B transmission electron microscope (JEOL, Tokyo, Japan) at an accelerating voltage of 80 kV.

### Growth experiments.

Physiological tests were performed in liquid, 2-fold-diluted NMS medium with methane. The growth of strain C50C1 was monitored by measuring its optical density at 600 nm (OD_600_) for 2 weeks under a variety of conditions, including temperatures of 2 to 37°C, pHs of 4.0 to 8.5, and NaCl concentrations of 0 to 4.0% (wt/vol). Variations in pH were achieved by mixing 0.1 M solutions of H_3_PO_4_, KH_2_PO_4_, K_2_HPO_4_, and K_3_PO_4_. The utilization of potential carbon sources was examined using 0.05% (wt/vol) concentrations of the following compounds: formate, glucose, sucrose, galactose, lactose, fructose, citrate, succinate, pyruvate, acetate, and ethanol. The ability to grow on methanol was tested in NMS medium containing 0.01 to 5% (vol/vol) methanol.

Nitrogen fixation activity was assessed by monitoring growth in nitrogen-free medium. Incubations were performed in batches in triplicates. Bottles of 120 ml were sterilized and aseptically supplied with 17 ml of liquid 5-fold-diluted sterilized ammonium mineral salts (AMS) medium or 5-fold-diluted nitrogen-free mineral salts (MS) medium (Table S1). The headspace contained either an ambient or low O_2_ atmosphere (2%, vol/vol). Low O_2_ concentrations in the headspace were achieved by 5 rounds of vacuum application to the bottles, followed by flushing with N_2_-CO_2_ (90%/10%, vol/vol). Subsequently, 2% (vol/vol) O_2_ was added aseptically. All bottles received 10% (vol/vol) CH_4_ aseptically. Prior to inoculation, biomasses from 3 batch incubations pregrown on 5-fold-diluted AMS, NMS, or MS medium to mid-exponential phase were pooled. Cells were washed twice to remove any remaining nitrogen source by pelleting the biomass in 50-ml tubes at 1,000 × *g* for 10 min (5810 centrifuge; Eppendorf, Hamburg, Germany). Subsequently, the supernatant was removed and replaced with nitrogen-free, 5-fold-diluted MS medium. Cells were dissolved in 5-fold-diluted MS medium. All bottles were inoculated with 3 ml of the washed cells at a starting OD_600_ of 0.05. The OD_600_ was measured using a spectrophotometer (Spectronic200; ThermoFisher Scientific, Waltham, MA, USA). The CH_4_ concentrations in the headspace were measured by injection of 50-μl gas samples into an HP 5890 gas chromatograph (Hewlett Packard, Palo Alto, CA, USA) equipped with a Porapak Q 100/120 mesh (Sigma-Aldrich, Saint Louis, MO, USA) and a flame ionization detector (FID). O_2_ concentrations were determined using an Agilent 6890 series gas chromatograph coupled to a mass spectrometer (Agilent, Santa Clara, USA) equipped with a Porapak Q column heated at 80°C, with helium as the carrier gas, as described previously (75).

### Molecular analyses.

Extraction, analysis, and identification of phospholipid-derived fatty acids (PLFAs), including dimethyl disulfide (DMDS) derivatization to determine double-bond positions, was performed as described by Dedysh et al. (19). DNA was extracted from 2 ml liquid culture using the PowerSoil DNA isolation kit (MO Bio Laboratories Inc., Carlsbad, CA, USA) according to the manufacturer’s protocol. The genomic DNA was sequenced on the Illumina MiSeq platform, with MiSeq reagent kit v3 (600 cycles, yielding 2× 300-bp paired-end sequencing reads; Life Technologies, Carlsbad, CA, USA). For genomic library preparation using the Nextera XT kit (Illumina, San Diego, CA, USA), in total 5 μl genomic DNA (gDNA) normalized to 0.2 ng/μl was used. Fragmentation was performed enzymatically, followed by incorporation of the indexing adapters and amplification of the library as described by the manufacturer. Purification of the amplified library was performed using AMPure XP beads, and the quality and size distribution of the library were checked using the Agilent 2100 Bioanalyzer and the high-sensitivity DNA kit (Agilent Technologies, Santa Clara, CA, USA). Fluorimetric quantitation of the library was performed by Qubit using the double-stranded DNA (dsDNA) HS assay kit (Thermo Fisher Scientific Inc., Waltham, USA). For normalization of the library, the concentration measured by Qubit and the average fragment size obtained with the Agilent 2100 bioanalyzer were used. After dilution to a 4 nM end concentration, the library was denatured and diluted according to the *MiSeq System Denature and Dilute Libraries Guide* (76) and loaded in the cartridge, and the sequence run was started using the Illumina MiSeq platform (Illumina, San Diego, CA, USA).

### Bioinformatic analysis.

Illumina raw sequencing reads were imported into CLC Genomics Workbench (v11.0.2; Qiagen/CLCbio, Aarhus, Denmark) and trimmed on the bases of quality and length (≥100 bp), resulting in nearly 11.5 million reads, which were used for subsequent analyses. Reads were assembled using CLC Genomics Workbench (assembly parameters were a word size of 20, a bubble size of 50, and a minimum contig length of 200; mapping parameters were a mismatch cost of 2, an insertion cost of 3, a deletion cost of 3, a length fraction of 0.5, and a similarity fraction of 0.8). As a slight contamination in the culture used for DNA extraction was observed, metagenomic binning was performed based on C+G content and sequencing depth (77). The assembled genome of strain C50C1 was composed of 42 contigs with an *N*_50_ of 199.476 bp, an overall genome size of 4.8 Mbp, and an average G+C content of 63%. Genome completeness and contamination were estimated by CheckM (78) to be 99.1% and 3.3%, respectively. Binned contig sequences were submitted to the RAST automated annotation pipeline (79), which includes genomic object prediction (CDSs and RNA genes), sequence homology searches, prediction of protein localization, and reconstruction of metabolic networks. Subsequently, the annotation was refined manually and compared to publicly available genomes of aerobic MOB.

### Data availability.

The high-quality draft genome of strain C50C1 is available at NCBI under BioProject accession number PRJNA361434.
