# Supplementary figures and images for: Methylotetracoccus oryzae Strain C50C1 Is a Novel Type Ib Gammaproteobacterial Methanotroph Adapted to Freshwater Environments
Source: mSphere. 2019 Jun 5;4(3):e00631-18. doi: 10.1128/mSphere.00631-18 (PMC6553558; doi:10.1128/mSphere.00631-18)

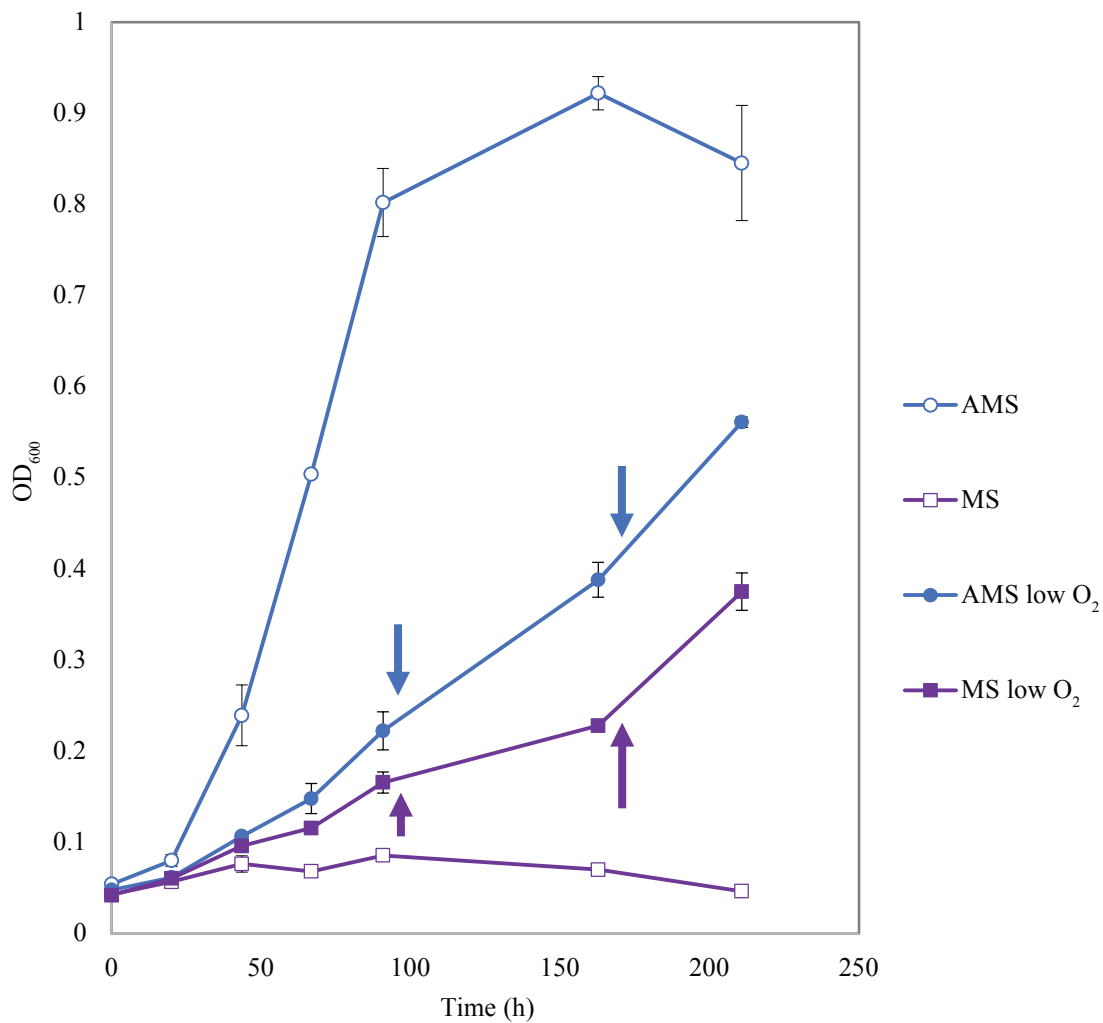

Supplement: FIG S1 [file mSphere.00631-18-sf001.pdf]

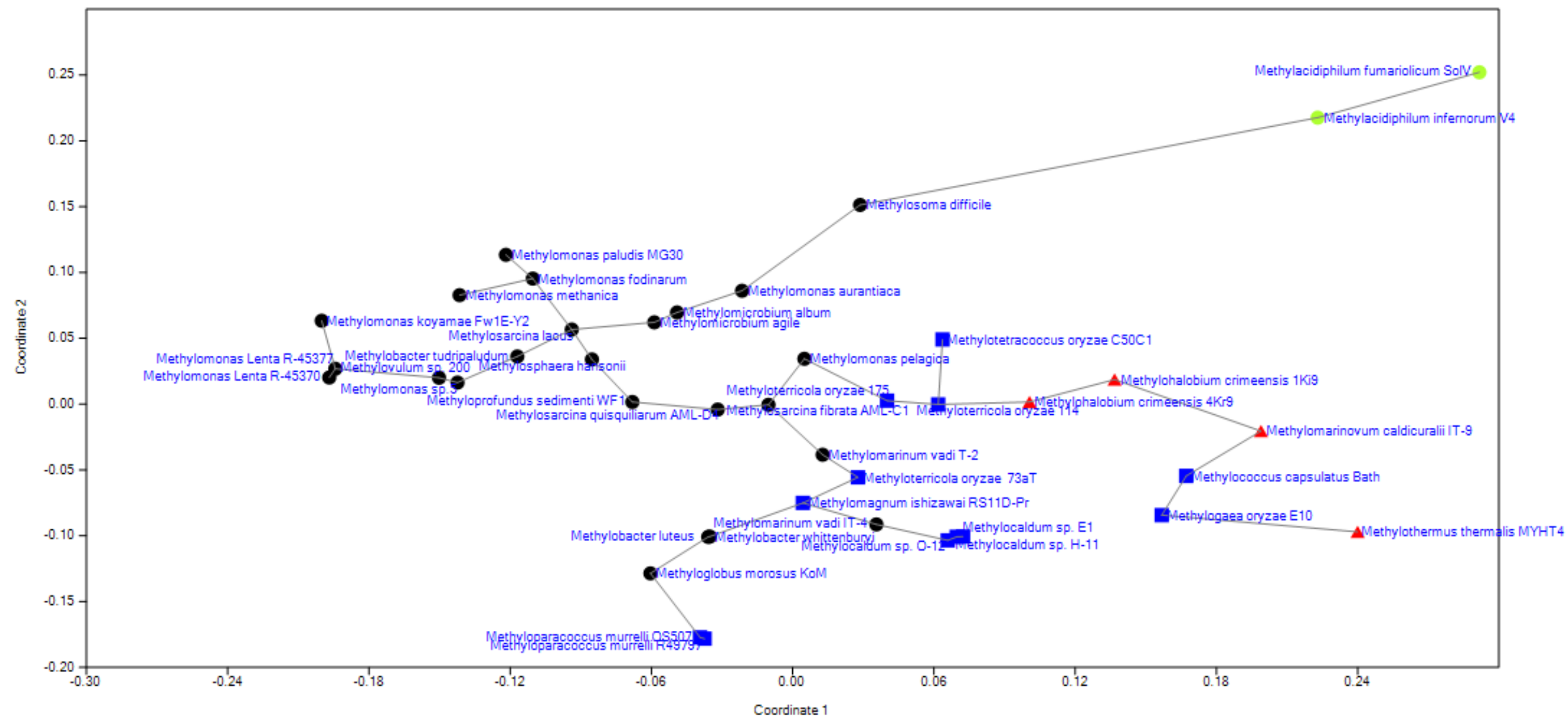

Supplement: FIG S2 [file mSphere.00631-18-sf002.pdf]
