# Supplementary material for: Methylotetracoccus oryzae Strain C50C1 Is a Novel Type Ib Gammaproteobacterial Methanotroph Adapted to Freshwater Environments
Source: mSphere. 2019 Jun 5;4(3):e00631-18. doi: 10.1128/mSphere.00631-18 (PMC6553558; doi:10.1128/mSphere.00631-18)
Supplement: TABLE S1 [file mSphere.00631-18-st001.docx]

| **Media composition** | **Final concentration (g/L)** |
| --- | --- |
| **AMS** | |
| KH_2_PO_4_ | 0.2 |
| MgSO_4_ × 7 H_2_O | 1 |
| CaCl_2_ × 2 H_2_O | 0.2 |
| NH_4_Cl | 0.5 |
| **NMS** | |
| KH_2_PO_4_ | 0.2 |
| MgSO_4_ × 7 H_2_O | 1 |
| CaCl_2_ × 2 H_2_O | 0.2 |
| KNO_3_ | 1 |
| **MS** | |
| KH_2_PO_4_ | 0.2 |
| MgSO_4_ × 7 H_2_O | 1 |
| CaCl_2_ × 2 H_2_O | 0.2 |
| **Trace element Solution 1** | |
| Trisodium Nitrilotriacetate (NTA) | 5 |
| FeSO_4_ | 5 |
| **Trace element Solution 2** | |
| Trisodium Nitrilotriacetate (NTA) | 4.5 |
| ZnSO_4_ × 7 H_2_O | 0.129 |
| CoCl_2_ × 6 H_2_O | 0.072 |
| MnCl_2_ × 4 H_2_O | 0.297 |
| CuSO_4_ × 5 H_2_O | 0.075 |
| NaMoO_4_ × 2 H_2_O | 0.066 |
| NiCl_2_ × 6 H_2_O | 0.057 |
| NaSeO_4_ × 10 H_2_O | 0.063 |
| H_3_BO_4_ | 0.042 |
| CeCl × 6 H_2_O | 0.072 |
