# Supplementary material for: Methylotetracoccus oryzae Strain C50C1 Is a Novel Type Ib Gammaproteobacterial Methanotroph Adapted to Freshwater Environments
Source: mSphere. 2019 Jun 5;4(3):e00631-18. doi: 10.1128/mSphere.00631-18 (PMC6553558; doi:10.1128/mSphere.00631-18)
Supplement: TABLE S2 [file mSphere.00631-18-st002.docx]

| **Substrate** | **OD_600_^*^** |
| --- | --- |
| Methane | 1.10 ± 0.15 |
| Methanol | 0.09 ± 0.01 |
| Acetate | 0.03 ± 0.005 |
| Pyruvate | 0.04 ± 0.01 |
| Succinate | 0.03 ± 0.005 |
| Malate | 0.03 ± 0.005 |
| Ethanol | 0.04 ± 0.005 |
| Glucose | 0.03 ± 0.005 |
| Fructose | 0.03 ± 0.005 |
| Sucrose | 0.03 ± 0.005 |
| Formate | 0.03 ± 0.005 |
| Formaldehyde | 0.02 ± 0.01 |
| control | 0.03 ± 0.005 |

**^*^**OD_600_ = maximal optical density reached.
