# Supplementary material for: Methylotetracoccus oryzae Strain C50C1 Is a Novel Type Ib Gammaproteobacterial Methanotroph Adapted to Freshwater Environments
Source: mSphere. 2019 Jun 5;4(3):e00631-18. doi: 10.1128/mSphere.00631-18 (PMC6553558; doi:10.1128/mSphere.00631-18)
Supplement: TABLE S3 [file mSphere.00631-18-st003.docx]

| **PLFA** | % of total PLFA | |
| --- | --- | --- |
|  | Average | SD^*^ |
| C14:0 | 0.34 | ±0.01 |
| C15:0 | 1.12 | ±0.06 |
| C16:0 | 17.73 | ±0.77 |
| C16:1w9t | 3.91 | ±0.05 |
| C16:1w9c | 33.01 | ±2.66 |
| C16:1w5t | 0.19 | ±0.02 |
| C16:1w7c | 18.13 | ±14.82 |
| C16:1w6c | 8.67 | ±0.03 |
| C16:1w5c | 5.95 | ±0.27 |
| C16:1 | 0.80 | ±0.02 |
| C17:0 | 0.15 | ±0.00 |
| C17:0 | 0.26 | ±0.03 |
| C17:1w8c | 0.16 | ±0.01 |
| C18:1 | 0.11 | ±0.02 |
| C18:0 | 0.53 | ±0.00 |
| C18:1w7c | 0.93 | ±0.18 |

^*^SD = standard deviation, calculated from two technical replicates.
